# Local dynamic stability as a responsive index for the evaluation of rehabilitation effect on fall risk in patients with multiple sclerosis: a longitudinal study

**DOI:** 10.1186/1756-0500-6-260

**Published:** 2013-07-09

**Authors:** Roger Hilfiker, Claude Vaney, Brigitte Gattlen, André Meichtry, Olivier Deriaz, Véronique Lugon-Moulin, Anne-Marie Anchisi-Bellwald, Cécilia Palaci, Denise Foinant, Philippe Terrier

**Affiliations:** 1 Institute Health & Social Work; HES-SO Valais-Wallis, University of Applied Sciences Western Switzerland, Sion and Leukerbad, Switzerland; 2 Berner Klinik Montana, Crans-Montana, Switzerland; 3 Institut de recherche en réadaptation- réinsertion (Institute for research in rehabilitation), Sion, Switzerland; 4 Clinique romande de réadaptation SUVACare, Sion, Switzerland; 5 RéSaR, Réseau d’études appliquées des pratiques de Santé, de Réadaptation/(ré)insertion, HES-SO, Delémont, Switzerland; 6 ZHAW, Zurich University of Applied Sciences, School of Health Professions, Institute of Physiotherapy, Technikumstrasse 71, Winterthur, 8401, Switzerland

**Keywords:** Gait, Maximal Lyapunov exponent, Accelerometer, Dynamic balance

## Abstract

**Background:**

Gait and balance problems are common in patients with multiple sclerosis, leading to high risk for falls. Local Dynamic Stability (LDS), a non-linear gait stability index, has been advocated as an early indicator of risk for falls. With this longitudinal study over three weeks, we aimed to assess the responsiveness of Local Dynamic Stability to a rehabilitation program and to compare it to other measures.

**Methods:**

Eighteen patients (mean 54 years, median EDSS score: 5) participated. They were admitted to inpatient rehabilitation and received a three weeks individually tailored program. They performed a 3-minute walking test at the beginning and at the end of the stay, as well as pain, wellbeing, fatigue, and balance assessment. The Local Dynamic Stability was computed from the acceleration signals measured with a 3D-accelerometer.

**Results:**

At the end of the rehabilitation process, patients reported reduced pain (Effect Size: −0.7), fatigue (ES:-0.6), and increased wellbeing (ES: 1.1). A small positive effect on static balance was observed (ES: 0.3). LDS was improved (ES: 0.6), and the effect was higher than walking speed improvement (ES: 0.4).

**Conclusions:**

The Local Dynamic Stability seemed responsive to assess rehabilitation effects in patients with multiple sclerosis. It could constitute a valuable gait quality index, which could evaluate potential effects of rehabilitation on fall risk.

**Trial registration:**

Current Controlled Trials ISRCTN69803702.

## Background

Gait problems are common in patients with multiple sclerosis (MS) and ranked by them as the most important bodily function [1]. There is evidence that patients with MS walk slower, with shorter steps, lower cadence, and less joint movement. Furthermore, they exhibit more variability in most gait parameters [2]. These might be some of the reasons for the high incidence of falls (52%) reported among people with MS [3]. In addition, patients with MS lose bone mass more rapidly than healthy persons matched for age- and gender [4]. As a result, MS is also associated with an increased risk of fractures [4,5].

Stability during walking (dynamic stability) can be defined as the ability to maintain functional locomotion despite the presence of external disturbances or internal control errors. An increased risk of falling is expected when patients are unable to appropriately execute avoidance strategies when facing unexpected obstacles or perturbations [6]. Accordingly, the largest perturbation that the locomotor system can withstand is referred to as “global stability” [6-8]. In parallel, humans permanently adjust gait parameters to compensate small perturbations that spontaneously occur from environmental (for instance uneven floor surfaces) and internal (neuro-control errors) sources: this is referred to as “local stability” [9,10].

Global stability can be analysed by pushing the locomotor system to the limits, for instance by inducing artificial trips while walking [11]. Conversely, Local Dynamic Stability (LDS) can be assessed during normal walking using Lyapunov exponents, which is a common technique to assess the divergence in pseudo-periodic processes [12]. In short, local divergence exponents (λ*) are computed to quantify how fast neighbouring trajectories of a reconstructed state space diverge. The rationale is that if motor control can efficiently manage small perturbations (low divergence), one can as well better cope with large perturbations that would lead to falling. Therefore, it has been suggested that LDS may be used as a fall predictor to differentiate fall-prone adults [13]. Furthermore, It has been shown that elderly are more locally unstable than young subjects [14]. In addition, by using a 3D dynamic models, two independent studies [7,8] suggested that λ* could be an early predictor for fall risk. Finally, the validity of LDS compared to other indicators has been recently discussed and LDS was found to be one of the best stability indexes [15].

In clinical practice as well as in fundamental research, walking limitations are important markers for the evaluation of the progression (i.e. improvement or deterioration) of the disease consequences. However, it remains unclear which parameters are best suited for the evaluation of the change in walking problems. Walking problems can get worse because of the natural course of the disease and can get better through interventions, as for example physiotherapy. Rehabilitation can reduce fatigue and enhance walking by improving force, movement patterns (efficiency of the gait-cycle), dynamic balance and self-efficacy [16-18]. Concerning LDS, It has been observed that fall prevention training programs could significantly improve gait stability in elderly people [19]. Although it has been recently shown that MS patients are less locally stable than healthy controls [20], the effect of therapeutic interventions has not been studied.

In a randomized controlled trial with 49 patients with MS, we recently evaluated whether Lokomat® (robot-assisted, body weight supported treadmill training) or walking training would have favourable effects on parameters such as quality of life, activity level, gait characteristics and perceived fatigue [21]. In the present analysis, we included the data of 18 patients with good gait capacity from this previous study to specifically explore whether the rehabilitation program would modify LDS. The aim of this secondary analysis was 1) to measure the LDS change after a three week inpatient rehabilitation setting, 2) to assess its responsiveness as compared to different other clinical measures, and 3) to evaluate whether LDS can be a clinically relevant gait quality index in this context.

## Methods

### Participants

The MS patients selected in the present analysis are from a previous randomized controlled trial with 49 participants. The details on inclusion criteria can be found in the published article [21]. In short, patients should have Kurtzke Expanded Disability Status Scale (EDSS) [22] score equal or higher than 3 and lower or equal than 6.5. The additional inclusion criterion for the present study was that patients should perform at least 2 × 34 continuous steps during a 3-minute walking test along a 90-meter corridor (one U-turn). Individuals who stopped repeatedly, or who walked very slowly, were excluded because a correct LDS assessment requires a minimal number of consecutive gait cycles. Eighteen patients met the inclusion criteria. Age, Gender, EDSS-Score for disease severity and other characteristics are reported in Table 1.

**Table 1:** Values at the beginning of the rehabilitation

**Variables**	**N**	**Mean**	**SD**	**Minimal**	**Max**	**p50**	**p25**	**p75**
Age	18	54	11	37	72	53	47	61
EDSS	18	5.11	1.27	3	6.5	5.5	4	6
Well Being	18	3.44	2.01	0	7	4	2	5
Rivermead	18	12.72	1.74	8	15	13	12	14
Berg Balance Scale	18	47.33	5.65	36	55	49	45	51
Spasticity	18	0.29	0.44	0	1.79	0.14	0.07	0.29
Pain	18	2.89	3.05	0	9	3	0	5
Physical Fatigue	18	20.06	6.75	3	30	20	18	24
Cognitive Fatigue	18	18.44	7.94	2	31	19	15	23
10-meter walk time (seconds)	18	13.39	5.37	7	26	11	11	15
3-min Walk distance (meters)	18	170.11	52.58	90	270	162	137	212
Speed 3-min walk test (m/s)	18	0.95	0.29	0.5	1.5	0.9	0.76	1.18
Step Frequency (Hz)	18	1.59	0.26	1.19	2.04	1.68	1.33	1.75
LDS: ML	18	0.34	0.06	0.21	0.46	0.35	0.29	0.38
LDS: V	18	0.36	0.07	0.23	0.48	0.36	0.30	0.40
LDS: AP	18	0.32	0.06	0.22	0.43	0.33	0.26	0.37

*N* number of participants without missing values, *SD* standard deviation, *Max* maximal values, *p50* median, *p25* 25th percentile, *p75* 75th percentile, *EDSS* The Kurtzke Expanded Disability Status Scale, *Hz* Hertz, *ML* medio-lateral, *V* vertical, *AP* antero-posterior.

The trial was performed in accordance to the Helsinki Declaration and was approved by the ethics committee of the Canton Valais, Switzerland. The trial was registered on http://www.controlled-trials.com/ISRCTN69803702. Written informed consent for participation in the study was obtained from all participants.

### Rehabilitation

Participants were admitted to a three-week inpatient rehabilitation. They received a semi- standardized, individually tailored rehabilitation program. One-half of the patients received in addition nine sessions of robotic assisted walking training, the other half nine session of normal walking in a group. In this sample, nine patients were in the robotic assisted walking training and nine patients in the normal walking group. More details can be found in the above-mentioned article [21].

### Dependent variables

Table 2 shows an overview of the variables used in the analysis and the corresponding references. The implementation of the methods is described in our previous article [21]. Here, we present in more details the method to assess gait stability based on acceleration signals.

**Table 2:** Measurements used in this study

**Name**	**Description**	**Outcome**	**Reference**
Wellbeing	Subject’s assessment of general wellbeing	Visual analog scale	[23]
Rivermead Mobility Index	Questionnaire and direct observation	0 (low mobility) to 15 (good mobility)	[24]
Berg Balance scale	Ability to perform 14 different tasks (sit, stand, reach, lean over, turn etc..)	0 (low balance) to 56 (good balance)	[25]
Spasticity	Limb response, Modified Ashworth Scale	0 (no increase in tone) to 4 (limb rigid in flexion or extension)	[26]
Pain	Subject’s assessment of overall degree of pain	0 (no pain) to 10 (high pain)	[27]
Fatigue (physical)	Questionnaire “Würzburger Erschöpfung bei MS”.	0 (no fatigue) to 32 (high fatigue)	[28]
Fatigue (cognitive)	Questionnaire “Würzburger Erschöpfung bei MS”.	0 (no fatigue) to 36 (high fatigue)	[28]
10-meter time	Short term preferred walking speed measured over 10 meter	Time	[29]
3-minute Distance	Maximal distance performed as fast as safely possible in 3-minutes	Distance	[29]
Cadence	Number of steps per unit of time, assessed by using accelerometric signal	Frequency (steps per second)	
Local Dynamic Stability	Gait stability by assessing average divergence of acceleration signals	3D divergence exponents (λ*)	See text

For a detailed prescription please see reference [21].

### Instrument

The inertial sensor was a tri-axial accelerometer: size 6.4 cm × 6.2 cm × 1.4 cm, weight 75 g (Dynaport (MiniMod) McRoberts BV, The Hague, The Netherlands). The sample rate was 100 Hz. Recorded signals were read out with AccRead Acquisition Software (McRoberts BV, The Hague, The Netherlands). The data analysis was performed with Matlab (Mathworks, USA).

### Walking trials

During the first and last week of the rehabilitation, patients walked three minutes as fast as safely possible in a 90-meter length corridor (one U-turn only allowed per trial). The triaxial accelerometer was attached to the lower back at the level of the third lumbar vertebra and measured trunk acceleration in mediolateral (ML), vertical (V) and anteroposterior (AP) directions.

### Data pre-processing

Forty-nine patients performed the 3-minute walking tests wearing the accelerometer. Each 3D- acceleration signal was graphically inspected: the goal was to select only individuals with sufficient consecutive strides (i.e. steady gait) to allow LDS assessment and meaningful intra- and inter-individual comparisons. In the acceleration signal, each step is clearly identified by a peak corresponding to the heel strike. For each test, two 34-step periods were selected (2 × 17 strides). The acceleration signals of 18 patients met the quality criteria and were kept for the subsequent analysis. Step frequency (SF) was assessed by using Fast Fourier Transform (FFT) of the vertical acceleration signal. Then, the signals containing the 17 strides (whose duration depends upon walking speed and SF) were time-normalized to a uniform length of 2500 samples using a polyphase filter implementation.

### Local dynamic stability

The method for quantifying the LDS by using largest Lyapunov exponent has been extensively described in the literature [9,10,30]. It examines structural characteristics of a time series that is embedded in an appropriately constructed state space. A valid state space contains a sufficient number of independent coordinates to define the state of the system unequivocally. The state space (or attractor) was reconstructed according to the Takens’ theorem, as classically applied in gait dynamics studies [9], and according to the latest recommendation in the field [31]. Embedding dimension and time delay were assessed by using respectively Global False Nearest Neighbors (GFNN) analysis and Average Mutual Information (AMI) function. Uniform time delay values (ML: 9, V: 11, AP: 15) were used according to the average AMI results. A constant dimension of 6 was set for all directions, according to the average GFNN results. The mean exponential rate of divergence of initially nearby points in the reconstructed space serves to compute logarithmic divergence diagrams [12]. The maximum finite-time Lyapunov exponents (λ*) were estimated from the slopes of linear fits in those divergence curves. Strictly speaking, because divergence curves are non-linear, multiple slopes could be defined and so no true single maximum Lyapunov exponent exists. The slopes (exponents) quantify local divergence (and hence local stability) of the observed dynamics at different time scale, and should not be interpreted as a classical maximal Lyapunov exponent in chaos theory. As a result, the term of “divergence exponent” is more appropriate. The Short-term divergence (short-term LDS) was estimated by fitting the divergence curve over a time scale ranging from a pair of nearest neighbours (initial perturbation at zero time) to the duration corresponding to one stride, (i.e. over the 147 first samples, given the resampling of the 17-strides signals to a uniform length of 2500 samples).

### Statistics

**Figure 1:**
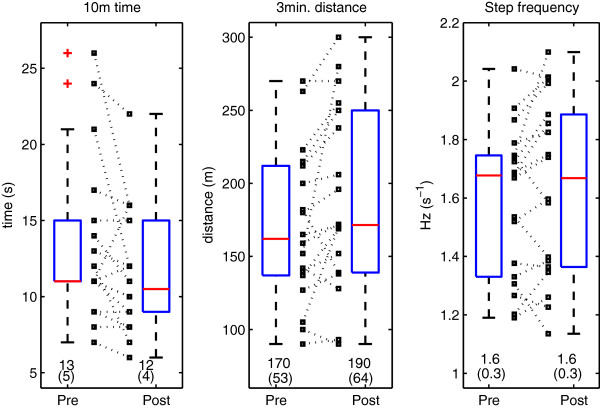
**Descriptive statistics of the gait-related variables.** Eighteen inpatients participated in a three week rehabilitation program. Their gait performance was evaluated at the beginning of the stay (pre) and at the end (post). The time they had to perform ten meters (10-meter time), the maximal distance walked during 3-minutes and the average walking cadence (step frequency) are presented as boxplots (median and quartiles), as well as scatter plots (small squares). Dotted lines link individual pre- and post-results. Values are mean and SD.

**Figure 2:**
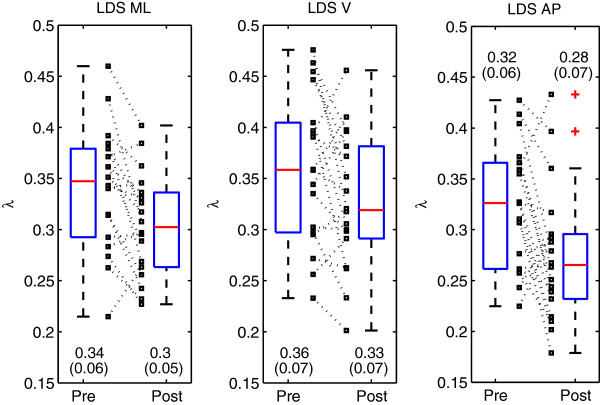
**Descriptive statistics of the local dynamic stability.** Eighteen inpatients participated in a three week rehabilitation program. Their local dynamic stability (LDS) was evaluated at the beginning of the stay (pre) and at the end (post) with a 3D motion sensor attached to the lower back, which measured trunk acceleration in the mediolateral (ML), vertical (V) and anteroposterior (AP) direction. The spread of the individual results are presented as boxplots (median and quartiles), as well as scatter plots (small squares). Dotted lines link individual pre- and post-results. Values are mean and SD.

Descriptive statistics (mean, standard deviation (SD), range, median, quartiles) of the dependent variables measured at the beginning of the stay (before the rehabilitation) were computed (Table 1). The spread of the individual results concerning gait performance (10-meter time, 3-minute distance, step frequency, Figure 1) and LDS (Figure 2) have been represented as boxplots and scatter plots for both pre-rehabilitation and post-rehabilitation conditions. The overall variance among subjects was described using the coefficient of variation (CV = SD/mean × 100).

In order to compare the responsiveness of the dependent variables (Table 2) to the rehabilitation program, we used the unbiased standardized effect size g (Hedges’s g), which is a variant of the Cohen’s d for inferential measures [32]. The precision estimates have been evaluated by computing 95% confidence intervals on g.

## Results

Among the recruited patients [21], the 18 patients, who exhibited sufficient continuous steps (2 × 34) were 12 women and 6 men. They were between 37 and 72 years old, with a mean age of 54 years. The mean EDSS score was 5 with a range from 3 to 6 points. The mean self-chosen walking speed during the 3-minute walking test was 0.95 meter per second, ranging from 0.5 to 1.5 meter per second (values between 1.2 and 1.5 m/s are considered as normal). See Table 1 for other characteristics of the participants.

The gait-related variables are described in Figure 1, before and after the rehabilitation program. A substantial variability among patients (CV) is observed: 10-meter time: pre 38%, post 33%; 3-minute distance: pre 31%, post 34%; step frequency (cadence): pre 19%, post 19%. LDS estimates are presented in Figure 2. They were globally more consistent among patients, namely, the variability (CV) was LDS ML: pre 18%; post 17%; LDS V: pre 19%, post 21%; LDS AP: pre 19%, post 25%. The relative change (i.e. (post – pre) / pre) was: ML −12%; V −8%; AP −13%. Note that negative values indicates a smaller divergence and hence an improved local stability.

### Responsiveness

**Figure 3:**
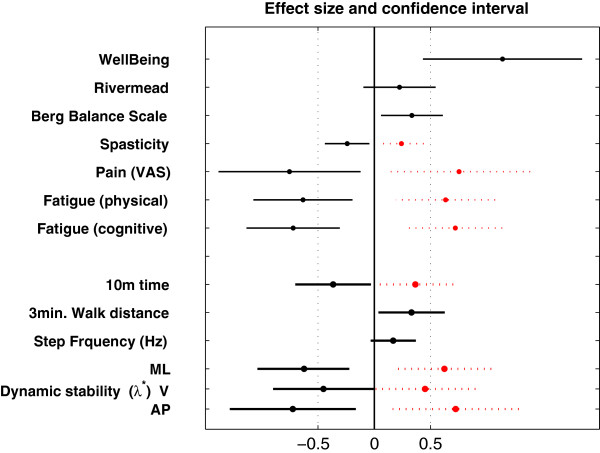
**Effect sizes with 95% confidence intervals.** The changes from the start to the end of the rehabilitation (difference = end minus start) was standardized by SD (unbiased effect size, Hedges’s g). Negative effect sizes were mirrored to the right (positive) side and plotted with intermittent red lines to allow better comparison between absolute effect sizes. Variables for which a higher value (positive effect size) would indicate a worse outcome are: Spasticity, Pain, Fatigue, 10-meter walking time, and local dynamic stability (LDS). Variables for which a lower value (negative effect sizes) would indicate a worse outcome are: Well-Being, Rivermead, Berg Balance Scale, 3-minute walking distance and step frequency. Bold lines indicate gait related variables.

The effect sizes (Hedges’ g) were, in order from largest to smallest (ignoring signs): 1.14 (95% CI 0.43 to 1.85) for the Wellbeing VAS, -0.75 (95% CI −1.38 to −0.12) for the pain VAS, -0.73 (95% CI −1.28 to −0.16) for the AP LDS, -0.72 (95% CI −1.14 to −0.31) for the cognitive subscore of the fatigue scale, -0.63 (95% CI −1.07 to −0.19) for the physical subscore of the fatigue scale, -0.62 (95% CI −1.04 to −0.21) for the ML LDS, -0.45 (95% CI −0.90 to −0.00) for the V LDS, -0.37 (95% CI −0.70 to −0.03) for the 10-meter walking time, 0.33 (95% CI 0.06 to 0.61) for the Berg Balance scale, 0.33 (95% CI 0.04 to 0.63) for the 3-minute walking distance, -0.24 (95% CI −0.44 to −0.04) for the spasticity scale (Ashworth), 0.22 (95% CI −0.10 to 0.55) for the Rivermead and 0.17 (95% CI −0.03 to 0.37) for the step frequency. From the balance and walking related measures, the antero-posterior local divergence exponent had the largest effect size (Figure 3).

## Discussion

With this secondary analysis of data from a trial in patients with Multiple Sclerosis during a three-weeks inpatient rehabilitation, it was observed that LDS was more sensitive to change than other gait performance indexes. Furthermore, a concomitant improvement in well-being, pain, fatigue and balance has been observed.

The strengths of this study are the longitudinal design and that several complementary measures were used, for example the Berg Balance Scale, Spasticity or the Rivermead Mobility Index and thus the responsiveness of the LDS can be compared.

The limitation of this study was that only 18 patients could be analysed and that the patients with more severe disease symptoms (e.g. EDSS over 6 points) were not included in our analyses because they did not exhibit steady gait during the 3-minute test.

Because inertial sensors (accelerometers) are small, non-invasive, reasonably cheap and easy to use, we [30,33] and others [34,35] have suggested that simple accelerometers and non-linear analyses could be used to assess gait quality in clinical setting. The study design was a balance between the need of sufficient consecutive strides to compute accurate local divergence exponent, the necessity to not expose patients to long and painful walking sessions, and the space limitation of the building [30]. It has been shown that reliable LDS estimates should be measured over more than 150 consecutive strides [36]. However, it seems that sufficient accuracy is also obtained by repeating short walking tests several times [37,38].

There is increasing evidence that LDS is a pertinent bio-marker for various diseases and conditions [39,40]. In particular, LDS was found to be lower in elderly [14,41]. Moreover, fall-prone elderly were found to exhibit significantly lower LDS, as compared to healthy counterparts [13]. By using Galvanic Vestibular Stimulation (GVS) in healthy subjects, Van Schooten et al. [42] concluded that LDS could be used to assess balance control in gait, and for the diagnosis of stability problems. As also recently suggested by others [20] LDS is a parameter, which is worth to be studied in MS patients in order to better assess gait quality and fall risks.

In the recent literature, there are studies that evaluate short-term LDS over one stride [10,30,43,44]. and other studies over one step (0.5 stride) [31,42,45]. The rationale behind such a methodological choice remains unclear. Here, we chose to compute LDS over one stride, hypothesizing that potential gait asymmetries (which are often present in MS patients) might bias LDS estimates computed over one step more than LDS estimates computed over one stride.

Recently, using the same methodology as in the present article, we observed that orthopaedic shoes improved LDS in patients with foot & ankle injuries (N = 25) [30]. The relative change induced by prescription footwear was: ML 10%, V 9% and AP 7%. The results of the present study show a slightly larger effect of the rehabilitation process (relative change: 8% - 13%), with the highest change in the AP direction. Given the small sample size (and hence large confidence intervals on ES), it is unclear whether the differences among the different axes and between both studies are physiologically relevant. For comparison, in a recent study that also used trunk accelerometry (N = 25), it has been observed that carrying heavy loads destabilizes gait (change in LDS +10%) in the AP direction only [44].

Our results indicate that standard rehabilitation over three weeks, including walking training either in a group or individually on a robotic assisted gait trainer, significantly enhance LDS, especially in antero-posterior and medio-lateral direction. The effect size indicates that the change is more likely practically relevant at group level. It is worth noting that traditional gait measures (10-meter time, 3-minute walk distance) showed lower effect sizes.

The observed changes in gait parameters may be due to a combination of multiple factors: 1) pain reduction, which may have enhanced confidence and gait control; 2) higher strength and lower spasticity of the lower limbs, which may have increased the control of the muscles on the joints; 3) better general coordination and awareness, which may have improved both static and dynamic stability. These hypotheses are supported by the significant change in the other parameters: the intervention had significantly reduced pain, fatigue and spasticity, and improved static balance [21]. However, because of the small sample size (N = 18), it was not reliable to assess the correlations between the variables. Therefore, further studies are needed to relate the improvement in dynamic stability with other clinical outcomes. There is evidence that resistance training (see e.g. [46]), endurance training [47], as well as combined training [48] can improve gait in patients with multiple sclerosis (see e.g. [46]), however, there is no evidence on the best modalities to improve gait stability.

Further research could evaluate more severely attained patients and compare the LDS with other non-linear indexes. Furthermore, it would be interesting to compare gait on different surfaces, e.g. tar, grass, or stones [49]. An important aspect to evaluate would be the association between LDS and subsequent falls. If associations can be shown in longitudinal studies, the index could be used to assess the immediate effect of interventions that aim to improve dynamic stability. Furthermore, larger studies should compare the local dynamic stability indexes with established balance measures, as for example the Berg Balance Scale or the Dynamic Gait Index. In our study, both the Berg Balance Scale and the local dynamic stability index showed high responsiveness, but they seem only moderately correlated (non-significant results, not shown).

## Conclusions

MS patients may improve their dynamic stability after a three-week rehabilitation program. This may indicate a lower falling risk, provided LDS is considered valid for this prediction. Furthermore, LDS seems more responsive to change than other classical gait performance index. In addition, other indexes (balance, spasticity, fatigue) exhibit concomitant enhancement that may explain the LDS improvement. This opens the perspective for a better evaluation of the effect of rehabilitation on gait in MS patients.

## Abbreviations

LDS: Local dynamic stability; EDSS: Kurtzke expanded disability status scale; ES: Effect size (Hedges’g); ML: Mediolateral; V: Vertical; AP: Anteroposterior; MS: Multiple sclerosis; λ*: local divergence exponents; GVS: Galvanic vestibular stimulation.

## Competing interests

The authors declare that there are no known conflicts of interest associated with this publication and there has been no significant financial support for this work that could have influenced its outcome.

## Authors’ contributions

RH conceived the study, analysed and interpreted the data, and drafted the manuscript. VC conceived the study, assessed data, interpreted the data and revised the manuscript. BG conceived the study, assessed the data, interpreted the data and revised the manuscript. AM performed data analyses, interpreted data and revised the manuscript. OD interpreted data and revised the manuscript. VLM, AMAB, CP and DF assessed the data and revised the manuscript. PT analysed and interpreted the data, drafted the manuscript and revised the manuscript. All authors read and approved the final manuscript.
